# Effects of Different Force Fields and Temperatures on the Structural Character of Abeta (12–28) Peptide in Aqueous Solution

**DOI:** 10.3390/ijms12118259

**Published:** 2011-11-21

**Authors:** Zanxia Cao, Lei Liu, Liling Zhao, Jihua Wang

**Affiliations:** 1 Shandong Provincial Key Laboratory of Functional Macromolecular Biophysics, Dezhou University, 566 University Rd. West, Dezhou 253023, China; E-Mails: qiayilai@mail.ustc.edu.cn (Z.C.); leiliusid@gmail.com (L.L.); zhaoll@sina.com (L.Z.); 2 Department of Physics, Dezhou University, 566 University Rd. West, Dezhou 253023, China; 3 Department of Computer Science and Technology, Dezhou University, 566 University Rd. West, Dezhou 253023, China

**Keywords:** Aβ (12–28) peptide, different force field, different temperature, molecular dynamics simulation, structural character, free energy surface

## Abstract

The aim of this work is to investigate the effects of different force fields and temperatures on the structural character of Aβ (12–28) peptide in aqueous solution. Moreover, the structural character of Aβ (12–28) peptide is compared with other amyloid peptides (such as H1 and α-syn12 peptide). The two independent temperature replica exchange molecular dynamics (T-REMD) simulations were completed by using two different models (OPLS-AA/TIP4P and GROMOS 43A1/SPC). We compared the models by analyzing the distributions of backbone dihedral angles, the secondary structure propensity, the free energy surface and the formation of β-hairpin. The results show that the mostly populated conformation state is random coil for both models. The population of β-hairpin is below 8 percent for both models. However, the peptide modeled by GROMOS 43A1 form β-hairpin with turn located at residues F19-E22, while the peptide modeled by OPLS-AA form β-hairpin with turn located at residues L17-F20.

## 1. Introduction

Alzheimer’s disease is the result of deposition of Aβ peptides [1–3]. In particular, the Aβ (12–28) peptide (the 12–28 fragment) is shown to have behavioral effects in mice [4,5], formation of fibril aggregates [6] and toxic effects *in vitro* [7]. In 2,2,2-trifluoroethanol(TFE) or membrane-mimicking environments [8], Aβ (12–28) peptide adopts α-helical conformation. However, the isolated Aβ (12–28) peptide in water co-exists among α-helix, polyproline (PPII), β-strand and random-coil structures according to several sources of experimental evidence [9,10].

The quality of molecular dynamic simulations for protein and peptide depends greatly on the accuracy of empirical force fields, water model and the detail of simulation [11–16]. Moreover, force fields are parameterized with special water model. It is necessary to select the possible combination of force field with water model carefully [17]. The effects of different force fields on the equilibrium structures of non-amyloid peptides have already been reported [18,19]. Matthes *et al.* [20] have presented a systematic study of sampling behavior and secondary structure formation with eight different molecular dynamics models (different force fields, different water models and different schemes for calculating electrostatic interactions). The results indicated that a number of distinct trends in the folding behavior, for example, AMBER99 force field favors helical structure, GROMOS96 [21] force field may overestimate β conformation, while the OPLS-AA force field [22] generates a better balance between α and β structure.

Many researchers [23–26] have done extensive studies on amyloid peptides using different force fields and different water models. For example, Daidone *et al.* [27] and Simona *et al.* [28] have revealed the structure of Aβ (12–28) peptide by using GROMOS96/SPC model, the β-hairpin with β-turn (F19-E22) was formed after 48ns at 320K. Baumketner *et al.* [29] have explored the folding landscapes of Aβ (12–28) peptide by using OPLS-AA/TIP3P model, the probability of β-hairpin conformation which possess a β-turn located at L17-F20 was 0.10. We note that the time scale of these simulations is much shorter than our time (300 ns/replica). The different force fields gave different results. Moreover, other amyloid peptide such as H1 peptide (residues 109–122 of the Syrian hamster prion protein) have been studied by conventional molecular dynamics simulation using the GROMOS force field and the SPC water model [27,30,31] and the simulation results were in good agreement with experiments for various β-peptides [32]. The effects of different force fields (the OPLS-AA and the GROMOS 43A1) with the SPC water model were compared for H1 peptide [33,34] using T-REMD [35]. The simulation using GROMOS 43A1 force field starting from α-helix sampled the conformation cluster close to β-sheet structure, and this cluster contained 39 percent of all the conformations. For the simulation using the OPLS-AA force field with the SPC [36] water model, the folded β-hairpin structure was more destabilized than those in the GROMOS 43A1 force field and experiments about some β-peptides (not the H1 peptide). Cao *et al*. [37,38] have investigated the structural and thermodynamics characters of α-syn12 peptide (residues 1–12 of the human α-synuclein protein) in aqueous solution by using GROMOS 43A1/SPC model, and the results showed that the isolated α-syn12 peptide in water adopted four different conformational states, one was β hairpin and contained 47 percent of all the conformations. It is interesting to know whether the three peptides (H1 peptide, α-syn12 peptide and Aβ (12–28) peptide) share the same structural and thermodynamics character in solution.

In this work, the effects of different force fields (OPLS-AA and GROMOS 43A1) and temperatures were compared by computing structural character of Aβ (12–28) peptide in aqueous solution using temperature replica exchange molecular dynamics (T-REMD). The two independent T-REMD simulations were completed starting from α-helix, respectively. Each replica was run for 300 ns, the total MD simulation time of all replicas was 10.8 *us*. The last 100 ns of the trajectory of 300 K spanning has been analyzed. The performance of each force field and temperature was assessed from the parameters such as the distributions of backbone dihedral angles, residue specific secondary structure propensity, formation of β-hairpin, and free energy surfaces. For both force fields, the peptide populated mostly random coil state. In the GROMOS 43A1/SPC model, the β-hairpin cluster which characterized by a turn located at residues F19-E22 and strands extending over residues H14-V18 and D23-N27 possess 4–7 percent of all conformations. However, for the simulation with the OPLS-AA/TIP4P model, the β-hairpin cluster, which is characterized by a turn located at residues L17-F20 and strands extending over residues H13-K16 and A21-D23, possess 4 percent of all conformations. The different force fields gave different β-hairpin, and we also note a strong structural dependence of our results on temperature. To our knowledge, this is the first report to study the effects of different force fields on the isolated Aβ (12–28) peptide in water by T-REMD.

## 2. Method

The two independent molecular dynamics simulations were completed starting from α-helix based on the GROMOS 43A1/SPC and the OPLS-AA/TIP4P force field [36,39,40] for Aβ (12–28) peptide. The Aβ (12–28) peptide (residues 12–28 of the Aβ (1–42) peptide responsible for Alzheimer’s disease, the sequence is VHHQKLVFFAEDVGSNK) was studied. Molecular dynamics simulations in the NPT ensemble were performed using the GROMACS software package [41]. The peptide is solvated in a rectangular box with the minimum solute-box boundary distance set to 1.0 nm. Protonation states of ionizable groups were chosen for neutral pH and the total charge of peptide was zero.

We used the particle-mesh Ewald method to treat the long-range electrostatic interaction with a grid spacing of 0.12 nm and a fourth order interpolation [42,43]. The temperature and pressure (setted as 1 atm) of the system was kept constant by using weak coupling algorithm [44]. The simulation using a temperature coupling time to external heat baths with a relaxation time of 0.1 ps and pressure coupling time of 0.5 ps, and using an isothermal compressibility of 4.575 × 10^−4^ (kJ mol^−1^ nm^−3^)^−1^. The time step for the MD integrator was set to 2 fs and SHAKE [45] was applied to constrain all bond lengths with a relative tolerance of 10^−4^.

The 36 replicas have been simulated at temperatures (in K) of 273, 275, 278, 280, 283, 285, 288, 290, 293, 295, 298, 300, 303, 306, 309, 312, 314, 317, 320, 322, 325, 328, 331, 334, 337, 340, 343, 345, 348, 351, 354, 357, 360, 363, 367 and 370 [46]. Each replica had been equilibrated at its respective temperature for 100 ps. Then 300 ns T-REMD simulations were performed for each replica, and replica exchanges attempted every 2 ps were based on the Metropolis criterion. Coordinates and energies have been recorded every 2 ps. The trajectories of 278 K, 298 K and 320 K spanning the last 100 ns (between 200 and 300 ns) have been analyzed. In order to verify the convergence of conformation sampling, the simulation time of GROMOS 43A1/SPC model increased to 400 ns for each replica. The distributions of backbone dihedral angles and conformation clusters which analyzed from 300 to 400 ns were compared with the results from 200 to 300 ns.

## 3. Results and Discussion

### 3.1. T-REMD Simulations

The ratios of successful exchange attempts were between 0.30 and 0.44 in these simulations, which is greater than 0.1, so the number of replicas in these simulation was sufficient.

### 3.2. Distributions of Backbone Dihedral Angles of Aβ (12–28) Peptide

**Figure 1:**
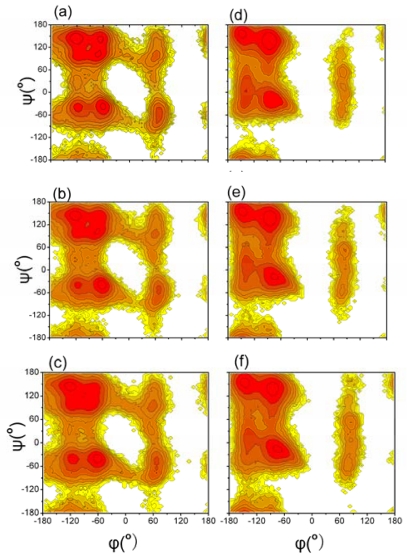
Potential of mean forces obtained from (ϕ, ψ) distributions of residues excluding glycine. Graph (**a–c**) plots the simulation using GROMOS 43A1/SPC model at 278 K, 298 K and 320 K respectively. Graph (**d–f**) plots the simulation using OPLS-AA/TIP4P model at 278 K, 298 K and 320 K respectively. Neighboring contour lines are separated by 2 kJ mol^−1^.

In order to investigate the influence of different force field and temperature on backbone conformations of the Aβ (12–28) peptide, we analyzed the distributions of the backbone (ϕ, ψ) angles for 13–27 residues except glycine. Data for these residues have been pooled together. The distributions of the Ramachandran (ϕ, ψ) angles of each residue excluding glycine were collected from the simulations with different models and temperatures, and potentials of mean force were computed (see Figure 1).

The distributions sampled by simulations with different models and temperatures were in general quite similar to each other. Different regions are defined as in the reference [47], α region: −180° < ϕ < 0° and −120° < ψ < 30°; Bridge region: −180° < ϕ < 0° and 30° < ψ < 90°; β region: −180° < ϕ < 0°, and 90^o^ < ψ < 180° or −180° < ψ < −120°. For the simulation with the GROMOS 43A1/SPC model, the probabilities of (ϕ, ψ) angles to fall into the α region at 278 K, 298 K and 320 K were 0.26, 0.28 and 0.30, respectively; the probabilities of (ϕ, ψ) angles to fall into the bridge region at 278 K, 298 K and 320 K were 0.06, 0.07 and 0.08, respectively; the probabilities of (ϕ, ψ) angles to fall into the β region at 278 K, 298 K and 320 K were 0.62, 0.58 and 0.55, respectively. The data in brackets were analyzed from 300 ns to 400 ns for the simulation with the GROMOS 43A1/SPC model. The probabilities were kept almost invariant at different simulation time. For the simulation with the OPLS-AA/TIP4P model, the probabilities of (ϕ, ψ) angles to fall into the α region at 278 K, 298 K and 320 K were 0.30, 0.33 and 0.34, respectively; the probabilities of (ϕ, ψ) angles to fall into the bridge region at 278 K, 298 K and 320 K were 0.06, 0.07 and 0.07, respectively; the probabilities of (ϕ, ψ) angles to fall into the β region at 278 K, 298 K and 320 K were 0.62, 0.58 and 0.56, respectively. The probabilities of (ϕ, ψ) angles to fall into the β and bridge regions were kept almost invariant with the different models at the same temperature (details in Table 1). However, the simulation with OPLS-AA/TIP4P model sampled more α region. With the increase of temperature, the simulation sampled more α region and less β region.

### 3.3. Residue Specific Secondary Structure Propensity of Aβ (12–28) Peptide

**Figure 2:**
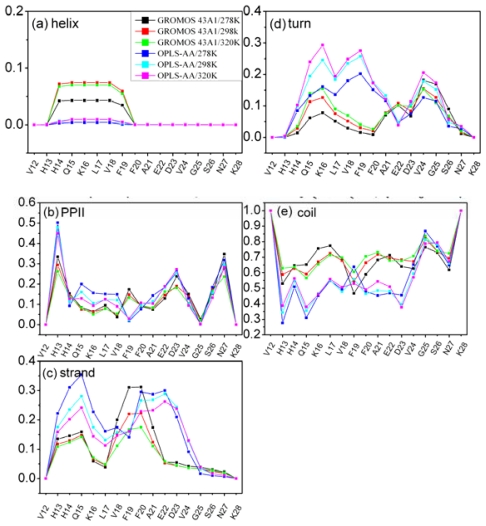
Residue specific secondary structure propensity of Aβ (12–28) with different models, at different temperatures. The PROSS protocol distinguishes five different types of secondary structure propensity.

Figure 1 shows that the simulations produced some sampling in the three different regions for both the two models. However, it is difficult to identify the secondary structure type of each residue based on the probabilities of the backbone (ϕ, ψ) angles. The residue specific secondary structure propensity of Aβ (12–28) were calculated with the program PROSS [48]. The results of our analysis for all 17 residues at different temperature and different models are shown in Figure 2. The program PROSS distinguishes five different types of secondary structure: α-helix, PPII, β-strand, β-turn and random coil. Aβ (12–28) peptide is seen to populate mostly random coil state. This result is consistent with the other experiments and simulation for Aβ [9,10,29,49]. Moreover, PPII, β-strand and β-turn structures are co-exist with coil in the two simulations.

The main differences between the two different models are as follows: (1) For the simulation with OPLS-AA/TIP4P model, the population of α-helix is vanishing. However, the population of α-helix is about 5 percent for the simulation with GROMOS 43A1/SPC model. (2) A high population of β-strand is observed for residues H13-K16 and F20-D23 (larger than 20 percent) for simulation with OPLS-AA/TIP4P model. These two β-strand regions are connected by a β-turn at L17-F19. However, there was no obvious trend to form β-strand for the simulation with GROMOS 43A1/SPC model.

The total amounts of secondary structure propensity at different temperatures and force fields are summarized in Table 2. The total amounts of secondary structure propensity are calculated as the sum of all residues. The percentage of β-strand and PPII structure is seen to decrease with the increase of temperature. In particular, the β-strand centered around H13-K16 and F20-E22 lose about 5 percent as the temperature is raised from 278 K to 320 K for the simulation with OPLS-AA/TIP4P model (Figure 2c). While the percentage of β-turn and coil is seen to increase with the increase of temperature. The general trend for β-turn in most residues is to increase with temperature (Figure 2d).

### 3.4. Free Energy Surface of Aβ (12–28) Peptide

**Figure 3:**
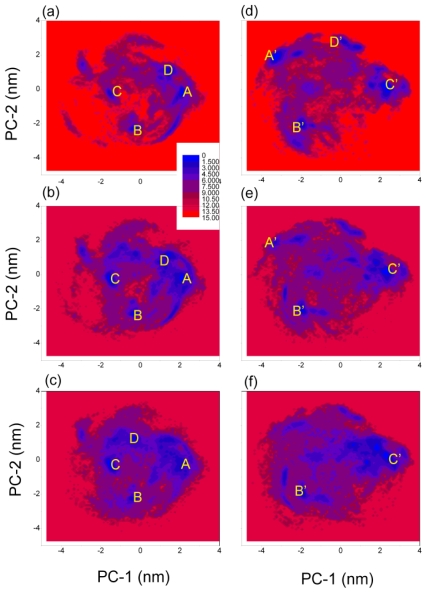
Free energy surfaces along the first two principle components (PC-1 and PC-2) in the simulation at (**a**) GROMOS 43A1/SPC and *T* = 278 K; (**b**) GROMOS 43A1/SPC and *T* = 298 K; (**c**) GROMOS 43A1/SPC and *T* = 320 K; (**d**) OPLS-AA/TIP4P and *T* = 278 K; (**e**) OPLS-AA/TIP4P and *T* = 298 K; (**f**) OPLS-AA/TIP4P and *T* = 320 K. Neighboring contour lines are separated by 2 kJ mol^−1^.

In order to analyze the structural characters of Aβ (12–28) peptide at different models and temperatures, the free energy surfaces (FESs) were constructed using two principle components (PC1 and PC2) as the reaction coordinates (see Figure 3). The programs “g_covar” and “g_anaeig” in the GROMACS package were used in the principal components analysis (PCA) [50]. The set of principal components are used as reaction coordinates to describe free energy surfaces at different models and temperatures. In the principal components analysis, only the fluctuations of all the backbone atoms of this peptide (51 atoms) were made use of. Here we choose the first two principle components (PC1 and PC2) as the reaction coordinates (see Figure 3). The free energy surfaces have obvious difference between the two models at different temperature. The lowest free energy is set as zero.

For the simulation with GROMOS 43A1/SPC model at 278 K, there were four highly populated regions on the PCA map centered near (2.05 nm, −0.25 nm), (−0.35 nm, −2.25 nm), (−1.55 nm, −0.15 nm) and (1.35 nm, 0.95 nm), and the four highly populated regions on the PCA map were similar for the simulation at *T* = 298 K. However, for the simulation at *T* = 320 K, the region centered near (1.35 nm, 0.95 nm) was vanished, and region centered near (−0.35 nm, 1.25 nm) was appeared (detail listed in Table 3). From the simulation at *T* = 278 K, we obtained the relative depths of the A, B, C and D minima that were 0.0, 1.4, 2.0 and 1.8 kJ mol^−1^, respectively; and in the simulation at *T* = 298 K, the corresponding depths were 0.5, 0.4, 0.0 and 0.9 kJ mol^−1^, respectively; and in the simulation at *T* = 320 K, the corresponding depths were 1.5, 1.5, 0.0 and 0.9 kJ mol^−1^, respectively. These results indicated that the lowest free energy region changed from (2.05 nm, −0.25 nm) to (−1.55 nm, −0.15 nm) with the increase of temperature. We obtained the local minima in the four regions: (**A**) located at (2.05 nm, −0.25 nm) of (PC1, PC2), which corresponds to conformation ensemble with a β-turn at position K16-F19, with strands extending over residues H14-Q15 and F20-A21; (**B**) located at (−0.35 nm, −2.25 nm), corresponds to conformation ensemble with a β-turn at position A21-D23, with strands extending over residues H14-L17 and V24-N27; (**C**) located at (−1.55 nm, −0.15 nm), corresponding to a random coil ensemble; (**D**) located at (1.35 nm, 0.95 nm), also corresponding to a random coil ensemble; another region located at (−0.35 nm, 1.25 nm) also corresponding to a random coil ensemble (shown in Figure 3).

For the simulation with OPLS-AA/TIP4P model at 278 K, there were four highly populated regions on the PCA map centered near (−3.25 nm, 1.95 nm), (−1.95 nm, −2.15 nm), (2.35 nm, 0.25 nm) and (0.25 nm, 2.75 nm). However, for the simulation at *T* = 298 K, the region centered near (0.25 nm, 2.75 nm) was vanished; for the simulation at *T* = 320 K, the regions centered near (0.25 nm, 2.75 nm) and (−3.25 nm, 1.95 nm) were vanished (detail listed in Table 3). From the simulation at *T* = 278 K, we obtained the relative depths of the A′, B′, C′ and D′ minima that were 0.0, 1.1, 1.0 and 1.6 kJ mol^−1^, respectively; and in the simulation at *T* = 298 K, the corresponding depths of the A′, B′, and C′ minima were 2.3, 0.7, and 0.0 kJ mol^−1^, respectively; and in the simulation at *T* = 320 K, the corresponding depths of the B′, and C′ minima were 1.4 and 0.0 kJ mol^−1^, respectively. These results indicated that the lowest free energy region changed from (−3.25 nm, 1.95 nm) to (2.35 nm, 0.25 nm) with the increase of temperature.

The corresponding representative structures are shown in Figure 3. We obtained the local minima in the four regions: (**A**′) located at (−3.25 nm, 1.95 nm) of (PC1, PC2), which corresponds to conformation ensemble with a β-turn at position V18-A21, with strands extending over residues H14-Q15 and S26-N27; (**B**′) located at (−1.95 nm, −2.15 nm), corresponding to a random coil ensemble; (**C**′) located at (2.35 nm, 0.25 nm), corresponding to conformation ensemble with a β-turn at position K16-V18, with strands extending over residues H13-Q15 and F19-A21; (**D**′) located at (0.25 nm, 2.75 nm), also corresponding to a random coil ensemble (shown in Figure 3).

### 3.5. Formation of β-Hairpin in the Aβ (12–28) Peptide

Although the above approach of using two dimensional reaction coordinates to visually represent the conformational space of peptides had been a simple and widely-used way, unavoidably, the free energy contour maps depend on the reaction coordinates. It is difficult to extract all structural information based on the two reaction coordinates. For example, the most frequently sampled conformation in the simulation using GROMOS96/SPC model by Daidone *et al.* was a β-hairpin characterized by a turn located at residues F19-E22. Moreover, in the simulation using OPLS-AA/TIP4P model by Baumketner *et al*., approximately 10 percent of the conformations were β-hairpin characterized by a turn located at residues L17-F20. However, we did not find a highly populated region corresponding to a β-hairpin characterized by a turn L17-F20 or F19-E22.

Can the simulations sample the β-hairpin conformation characterized by a turn L17-F20 or F19-E22? One way to reveal this question was to cluster them based on their mutual root-mean-square deviations of alpha-carbon positions (RMSD_Cα_). For the two independent simulations, we chose the conformations of the 278 K, 298 K and 320 K replicas to study. A total of 10,000 conformations from the last 100 ns trajectory were clustered based on their pair-wise RMSD_Cα_. The criteria of clustering are that the conformations are in the same cluster when RMSD_Cα_ is less than 0.15 nm among the conformations of this cluster, vice versa. In addition, all the conformations in the same cluster should be connected by the RMSD_Cα_ criteria. By the clustering criteria, conformations in the simulations fall into clusters of all kinds of sizes.

For the simulation with the GROMOS 43A1/SPC model, the representative conformation of one of the clusters was a β-hairpin characterized by a turn located at residues F19-E22 and strands extending over residues H14-V18 and D23-N27 (this structure is referred to as β-hairpin1). The probabilities of conformations fall into this cluster at 278 K, 298 K and 320 K were 0.07, 0.05 and 0.04, respectively.

For the simulation with the OPLS-AA/TIP4P model, the representative conformation of one of the clusters was a β-hairpin characterized by a turn located at residues L17-F20 and strands extending over residues H13-K16 and A21-D23 (this structure is referred to as β-hairpin2). The probabilities of conformations fall into this cluster at 278 K, 298 K and 320 K were all the 0.04. The PC1 and PC2 value of the conformations in this β-hairpin cluster were diverse, which was the reason we did not find a highly populated region on the free energy surfaces.

**Figure 4:**
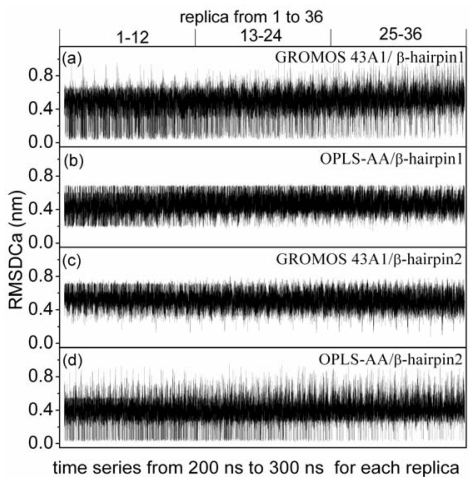
Positional root mean square deviations (RMSD) of alpha-carbon atoms with respect to the two different β-hairpin structures *versus* time series from 200 ns to 300 ns for each replica. Graphs (**a**) correspond to the simulation under GROMOS 43A1/SPC model with respect to the β-hairpin1 structure; Graphs (**b**) correspond to the simulation under OPLS-AA/TIP4P model with respect to the β-hairpin1 structure; Graphs (**c**) correspond to the simulation under GROMOS 43A1/SPC model with respect to the β-hairpin2 structure; Graphs (**d**) correspond to the simulation under OPLS-AA/TIP4P model with respect to the β-hairpin2 structure.

The different force fields gave different β-hairpin. In order to see whether the simulations sampled these two different β-hairpin, the positional root mean square deviations (RMSD) of alpha-carbon atoms (see Figure 4) from the two different β-hairpin structures were computed for the conformations of all replicas. For simulation under GROMOS 43A1/SPC model, Figure 4a shows the RMSD of alpha-carbon atoms with respect to the β-hairpin1 structure, the simulation can sample many conformations close to the β-hairpin1 structure, the lowest value is 0.04 nm. However, β-hairpin2 structures are not observed at any temperatures (see Figure 4c). For simulation under OPLS-AA/TIP4P model, Figure 4d shows the RMSD of alpha-carbon atoms with respect to the β-hairpin2 structure, the simulation can sample many conformations close to the β-hairpin2 structures, the lowest value is 0.03 nm. However, β-hairpin1 structures are not observed at any temperatures (see Figure 4b).

β-turn is an important factor involved in the folding of β-hairpin structure. To further describe the formation of β-turn in solution, the free energy profiles were calculated as a function of the distance between the hydrogen and oxygen atom involved in the β-turn hydrogen bonds. The relative free energy can be easily obtained by the following Equation:

$$\Delta F_{\text{state}1\to \text{state}2}=F_{state2}-F_{state1}=-RT\text{ln}(P_{state2}/P_{state1})$$

**Figure 5:**
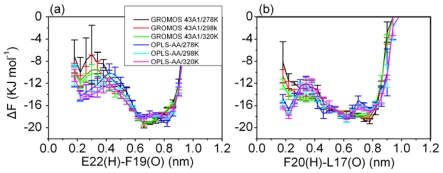
The free energy profiles were calculated as a function of the distance between the hydrogen and oxygen atom involved in the β-turn. The profiles were plotted as the distance between (**a**) E22 (H) and F19 (O); (**b**) F20 (H) and L17 (O). Error bars correspond to a standard deviation of the corresponding property as being obtained by considering four subsets of the simulation.

Where R is the universal gas constant, T is the temperature and *P*_state1_ and *P*_state2_ are the probabilities of being in distance 1 and 2, and the zero point of the free energy profile is set as zero. The profiles show large difference (shown in Figure 5) from the two different models. The OPLS-AA/TIP4P model sampled more conformations with less distance between the hydrogen and oxygen atom involved in the β-turn hydrogen bonds.

### 3.6. Solvent Exposure of Hydrophobic Residues in the Aβ (12–28) Peptide

**Figure 6:**
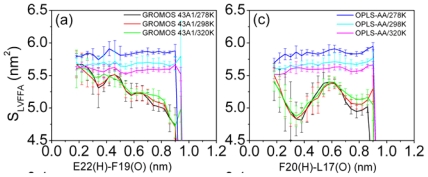

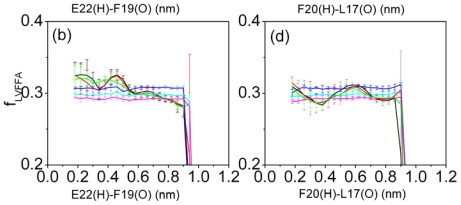
Solvent exposure of hydrophobic residues (LVFFA) as a function of the distance between the hydrogen and oxygen atom involved in the β-turn. Graphs (**a,c**) correspond to the solvent exposure of hydrophobic residues, S_LVFFA_. Graphs (**b,d**) correspond to the fraction of the solvent accessible surface of S_LVFFA_ to the total solvent accessible surface area (f_LVFFA_).

To further describe the structural character of Aβ (12–28) peptide in solution, the solvent accessible surface area of hydrophobic residues (LVFFA) as a function of the distance between E22 (H) and F19 (O) (or between F20 (H) and L17 (O)) were calculated (see in Figure 6). Error bars correspond to a standard deviation of the corresponding property were obtained by considering the four subsets of the simulation (the total 100 ns trajectory set has been evenly divided into the four 25 ns blocks for the simulation).

For the GROMOS 43A1/SPC model, the state has less distance between E22 (H) and F19 (O) is characterized by high solvent exposure of hydrophobic residues (S_LVFFA_ plotted in Figure 6a) and high fraction of the S_LVFFA_ to the total solvent accessible surface area (f_LVFFA_) (f_LVFFA_ plotted in Figure 6b). These results are consistent with the other molecular dynamics simulation results [27]. However, high solvent exposure of hydrophobic residues is observed for all conformations sampled using the OPLS-AA force field (shown in Figure 6c,d). There is no obvious difference of exposure of the hydrophobic residues between the folded and the unfolded structure in the simulation using the OPLS-AA force field.

## 4. Conclusions

Extensive evidence has been accumulated in recent years that several protein conformational diseases (for example: Alzheimer’s disease, prion-related disorders, Parkinson’s disease, *etc.*) have the same molecular basis: conformational change from a prevailing α-helical structure to β-sheet-rich. The amyloid peptides, such as Aβ (12–28) peptide, H1 peptide and α-syn12 peptide, have gained extensive attention in recent years. Experimental research has shown that the H1 peptide in water adopts a β-sheet structure according to several sources of experimental evidence [51,52], Aβ (12–28) peptide co-exists among PPII, β-hairpin and random coil structure. However, it is difficult to obtain the structural and thermodynamics characters of these peptides monomer in aqueous solutions by using experimental methods. Molecular dynamics simulation is a valid method for investigating this information. Generally, the quality of molecular dynamic simulations for proteins and peptides depends greatly on the accuracy of empirical force fields and water model. Our early studies have compared the effects of different force fields (the OPLS-AA and the GROMOS 43A1) with the SPC water model for H1 peptide using T-REMD, the results indicated that simulation using GROMOS 43A1 force field starting from α-helix sampled the conformation cluster close to β-sheet structure, and this cluster contained 39 percent of all the conformations. However, the folded β-hairpin structure was more destabilized by using OPLS-AA force field. Our other studies showed that the isolated α-syn12 peptide in water adopted four different conformational states, and one was β hairpin and contained 47 percent of all the conformations. The difference between these two force fields was obvious for these two amyloid peptides. Was the data arising from these researches biased by the intrinsic tendency of the GROMOS 43A1 force field?

In this study, the effects of different force field models were compared based on the computed structural characters of the Aβ (12–28) peptide. The two simulations populate mostly random coil state, and this result is consistent with the other experiments. In the GROMOS 43A1/SPC model, the β-hairpin cluster, which is characterized by a turn located at residues F19-E22 and strands extending over residues H14-V18 and D23-N27, possess 4–7 percent of all conformations. However, for the simulation with the OPLS-AA/TIP4P model, the β-hairpin cluster, which is characterized by a turn located at residues L17-F20 and strands extending over residues H13-K16 and A21-D23. possess 4 percent of all conformations. The above results show that the different force fields gave different β-hairpin. Moreover, the lowest free energy region changed with the increase of temperature. On the other hand, these results also indicated that the GROMOS 43A1 force field has no significant tendency to form a β-hairpin structure.

## Figures and Tables

**Table 1:** The respective probabilities for (ϕ, ψ) angles to fall within different regions at different temperatures and force fields. For the simulations under GROMOS 43A1/SPC model, the data in brackets were analyzed from 300 ns to 400 ns.

	GROMOS 43A1/SPC *T* = 278 K	GROMOS 43A1/SPC *T* = 298 K	GROMOS 43A1/SPC *T* = 320 K	OPLS-AA/TIP4P *T* = 278 K	OPLS-AA/TIP4P *T* = 298 K	OPLS-AA/TIP4P *T* = 320 K
α region	0.26 (0.26)	0.28 (0.27)	0.30 (0.29)	0.30	0.33	0.34
Bridge region	0.06 (0.07)	0.07 (0.08)	0.08 (0.08)	0.06	0.07	0.07
β region	0.62 (0.61)	0.58 (0.59)	0.55 (0.57)	0.62	0.58	0.56

**Table 2:** The total amounts of secondary structure propensity of Aβ (12–28) at different temperatures and force fields.

	GROMOS 43A1/SPC *T* = 278 K	GROMOS 43A1/SPC *T* = 298 K	GROMOS 43A1/SPC *T* = 320 K	OPLS-AA/TIP4P *T* = 278 K	OPLS-AA/TIP4P *T* = 298 K	OPLS-AA/TIP4P *T* = 320 K
α-helix	0.01	0.02	0.02	0.00	0.00	0.00
PPII	0.13	0.12	0.11	0.15	0.14	0.13
β-strand	0.10	0.08	0.07	0.16	0.15	0.14
β-turn	0.06	0.06	0.07	0.09	0.12	0.13
coil	0.70	0.71	0.72	0.59	0.59	0.60

**Table 3:** Coordinates and relative depths of the effective free energy surface minima of Aβ (12–28) peptide at different temperatures.

	(PC-1, PC-2) coordinates (nm) and relative depths (kJ mol^−1^) of the minima			
	
	A	B	C	D
GROMOS 43A1/SPC	(2.05, −0.25)	(−0.35, −2.25)	(−1.55, −0.15)	(1.35, 0.95)
*T* = 278 K	0.0	1.4	2.0	1.8
GROMOS 43A1/SPC	(2.15, −0.15)	(−0.35, −2.25)	(−1.45, −0.35)	(1.45, 1.15)
*T* = 298 K	0.5	0.4	0.0	0.9
GROMOS 43A1/SPC	(2.15, −0.25)	(−0.45, −2.25)	(−1.55, −0.25)	(−0.35, 1.25)
*T* = 320 K	1.5	1.5	0.0	0.9

	**A**′	**B**′	**C**′	**D**′

OPLS-AA/TIP4P	(−3.25, 1.95)	(−1.95, −2.15)	(2.35, 0.25)	(0.25, 2.75)
*T* = 278 K	0.0	1.1	1.0	1.6
OPLS-AA/TIP4P	(−3.35, 1.85)	(−1.95, −2.05)	(2.35, 0.25)	
*T* = 298 K	2.3	0.7	0.0	
OPLS-AA/TIP4P		(−1.95, −1.95)	(2.45, 0.15)	
*T* = 320 K		1.4	0.0	
